# Do Patients with Major Non-Communicable Diseases Receive Advice on Health Behaviors from Healthcare Professionals in the Gaza Strip, Palestine?

**DOI:** 10.4314/ejhs.v32i1.6

**Published:** 2022-01

**Authors:** Ahmed Hassan Albelbeisi, Ali Albelbeisi, Abdel Hamid El Bilbeisi, Amany El Afifi

**Affiliations:** 1 Department of Health Management and Economics, School of Public Health, Tehran University of Medical Sciences, International Campus (TUMS-IC), Tehran, Iran; 2 Medical Services Directorate, Gaza Strip, Palestine; 3 Department of Health Research, Ministry of Health, Palestine; 4 Department of Nutrition, School of Medicine and Health Sciences, University of Palestine, Gaza Strip, Palestine; 5 Faculty of Pharmacy, Al Azhar University of Gaza, Palestine

**Keywords:** Noncommunicable diseases, package of essential noncommunicable diseases (PEN), primary healthcare

## Abstract

**Background:**

NCDs tend to be long-term and are caused by genetic, physiological, environmental, and behavioral factors. Currently, NCDs account for 71% of deaths globally. The current study aimed to explore whether patients with NCDs receive advice on health behaviors from healthcare professionals in the PHCs in the Gaza Strip, Palestine.

**Methods:**

This research applied a cross-sectional survey design in five PHCs from October 2019 to December 2019, with 360 patients selected using a convenience sampling technique. A structured questionnaire on sociodemographic, history and patients' views on receiving advice on health behaviors from health care professionals was developed and collected. Data were analyzed using descriptive analyses and a One-way ANOVA test through SPSS-v22.

**Results:**

The patients reported receiving advice as follows: in terms of regular physical activity (54.5% ±13.6), in terms of eating a heart-healthy diet (49.3% ±11.5), in terms of treatment adherence (86.1% ±8.1), and in terms of stop tobacco for smokers' patients (43.9% ±16.8). Statistically significant differences were found between the five Gaza strip governorates in terms of regular physical activity, eating heart-healthy, and treatment adherence (P values <.05 for all).

**Conclusion:**

Participants claimed that the vast majority of them had received advice from healthcare professionals regarding attending regular follow-up and treatment adherence. In contrast, participants reported receiving advice from health care professionals regarding regular physical activity, eating a healthy diet, and stopping tobacco are suboptimal. There is a need to develop a strategy to ensure that healthcare professionals are committed to providing advice on health behaviors.

## Introduction

Non-communicable diseases (NCDs), or chronic diseases, tend to be long lasting and caused by genetic, physiological, environmental, and behavioral factors (1, 2). It is estimated that the four types of NCDs (cardiovascular diseases, cancer, diabetes, and chronic respiratory diseases) were responsible for 41 million deaths yearly and account for 71% of all deaths worldwide, most of NCDs death (74%) and the majority of premature deaths (82%) occur in low- and middle-income states (3). They share common behavioral risk factors involving tobacco use, unhealthy diet, physical inactivity, and alcohol use (3). Recently, there is an extensive scientific agreement that these factors contribute significantly to NCDs morbidity and mortality (4). Palestine is undergoing a rapid epidemiological transition, with growing the burden of NCDs. It is estimated that approximately two-thirds of elderly Palestinians suffering from NCDs.(5) Gaza Strip is a small part of the Occupied Palestinian Territory that is characteristic high densely populated territory. The total number of inhabitants is around two million, with more than 70% of them recorded as refugees, it is estimated that the prevalence of overweight and obesity among the Palestinian population was 62.4%, 24.4% respectively (6, 7). Gaza Strip since 2007 under siege that influencing the whole aspects of life, with more than half of the population suffering from poverty (8). In 2016, cardiovascular diseases remained the first leading cause of death among Palestinians, accounting for 30.6% of deaths recorded; cancer was the second leading cause of death, with 14.0% of deaths; complications of diabetes came in the fourth rank with a proportion of 8.0% (9). The WHO has established a package of essential NCDs interventions (WHO-PEN) that is a minimum set of interventions to address major NCDs in primary health care. The interventions are for the detection, diagnosis, treatment, and care of the main four types of NCDs (2). The Palestinian Ministry of Health introduced the WHO-PEN in PHCs and has delegated most NCDs management responsibilities to general physicians and nurses (10).

Besides, WHO-PEN protocol 2 is an essential tool in the implementation process, which is concerned with health education and counseling on healthy behaviors. The main instructions including progressively increasing physical activity to moderate levels; at least 150 minutes per week, restricting salt to less than 5 grams per day, five servings of fruits and vegetables per day, strongly advising all smokers to stop smoking and support them in their efforts, teach the patient how to take prescribed medications at home, and check the patient's understanding (11, 12). In Gaza Strip, a recent study demonstrated that more than half (57.7%) of PHCs had all of the counseling services recommended by the WHO-PEN protocol 2 (10). Another study conducted showed that 87.5% of health care professionals claimed that they are sometimes or always adherence to counseling on healthy behaviors in terms of physical activity, 80.5% in terms of a healthy diet, 54.8% % in terms of stop tobacco, and 82.7% in terms of attending medical follow-up based on WHO-PEN protocol 2 (13). Besides, another study conducted among patients with NCDs in Gaza Strip showed that 50.1% claimed that they are committed to healthy behaviors in terms of physical activity and 44.04% in terms of o a healthy diet (14). There are a lot of studies concerned with professionals' adherence to advice on healthy behaviors from the health professionals' perspective, but little is known whether patients with non-communicable diseases receive advice on health behaviors from healthcare professionals. The current study aimed to explore whether patients with noncommunicable diseases receive advice on health behaviors from healthcare professionals in the PHCs in the Gaza Strip, Palestine.

## Methods

**Study designs:** This cross-sectional study conducted from October 2019 to December 2019 in Gaza Strip, Palestine, used a convenience sampling technique.

**Study settings:** The study was conducted in five government primary health care centers. The participants were selected from the five Gaza Strip governorates based on the population density. The Gaza Strip is divided into five governorates, including North Gaza, Gaza, Deir Al Balah, Khan-Yunis, and Rafah.(15) The five PHCs were selected purposively to include, the centers with high utilization and attendance of NCDs services in each governorate of Gaza Strip, Palestine.

**Participants:** The study population was patients with non-communicable diseases receiving care at the five selected primary health care centers.

Patients with one of the major NCDs (cardiovascular disease, chronic respiratory disease, cancer, and diabetes), both genders, aged ≥ 18, registered and receiving treatment in the five selected MOH-PHCs in Gaza Strip were taken as inclusion criteria. And the exclusion criteria were patients who did not receive treatment in the five selected PHCs or their medical files are not available, aged < 18 years old.

**Sample size**: The traditional equation (Cochran) was used to calculate the sample size, the estimated sample size according to the equation is 384 cases, with a margin of error of 5% and confidence level of 95%.(16) *P* assumed 0.5. Besides, cases were distributed based on the population density, the distribution of participants was 19.4%, 34.4%, 14.4%, 19.5%, and 12.3% for North Gaza, Gaza City, Deir Al Balah, Khan Younis, and Rafah, respectively (15).

**Data collection**: A structured questionnaire was used to collect data from patients with NCDs via individual interviews during clinics' work hours. Data were collected by five data collectors after one full day of training about the study scope and purposes, questionnaire items, and possible areas for misunderstanding.

**Study instrument**: The initial questionnaire includes ten items to assess socio-demographic characteristics, fourteen items that explore whether a patient with non-communicable diseases receives advice on health behaviors from healthcare professionals within the past 12 months. For smoker patients, additional two items were included. The health behaviors recommendations are defined based on WHO-PEN protocol 2 for all NCDs patients (12). A five-point Likert scale was used for response categories with the rating scale of “always”, “often”, “sometimes”, “rarely”, and “never”.

**Translation and validation of the questionnaire**: Cross-cultural guideline process used in the translation of the questionnaire (17). Face and content validity were checked for the final Arabic draft questionnaire, independently validated by nine experts (researchers, health experts, nurses, and family doctors). The content validity index was calculated to rate the relevance of the questionnaire items (18). All items were rated as relevant. Minor changes in the language and the construction were did. Then, the questionnaire was piloted among 30 of the eligible NCDs patients, the results of the pilot study showed a good overall Cronbach's alphas of .78.

**Data analysis**: The SPSS software version 22 was used for the statistical analysis. Characteristics of the sample were described by descriptive statistics. Frequencies and percentages were used to described categorical variables, whereas Mean percentages and standard deviations (SD) were used to represent continuous variables. A One-way ANOVA test was used to compare means between the five Gaza strip governorates. P-values of less than 0.05 were considered statistically significant.

**Ethics approval and consent to participate:** The study protocol was approved by the Palestinian Health Research Council (Helsinki Ethical Committee of Research PHRC/HC/599/19). Second, official letters were obtained from the MoH-General Directorate of Human Recourses Development. In addition, orally informed consent was also obtained from each participant.

## Results

Three hundred and sixty patients with noncommunicable diseases agreed to participate in the study during the data collection period with a response rate of 93.7%. The study sample was distributed in each governorate as follows: (North Gaza, Gaza City, Deir Al Balah, Khan Younis, and Rafah) 71, 124, 52, 69, and 44 patients respectively.

**Characteristics of the Study Participants**

A total of 360 patients with NCDs were included in this study. The characteristics of the study participants are summarized in table 1. Aged (52. 6±9.9) years old, 23.5% over 60 years old. More than half (57.5%) of the study participants were female. The vast majority (75.3%) of participants were married, only 21.3% smokers, (51,1%) had at least a university degree, (43.3%) jobless or retired, about four-fifths had incomes less than 2000 NIS per month. Around 92% of the participants had DM, HTN, or both, 57.5% complain of NCDs less than five years. All study participants claimed that they did not use alcohol.

**Table 1:** Characteristics of the study participants (n=360)

Variables	NCDs patients	Percent
**Gender**		
Male	153	42.5
Female	207	57.5
**Governorate**		
North Gaza	71	19.7
Gaza	124	34.4
Deir Al Balah	52	14.4
Khan Younis	69	19.2
Rafah	44	12.2
**Age (Mean±SD: 52. 6±9.9)**		
≤ 40 years	46	12.8
41 to 60 years	229	63.6
> 60 years	85	23.6
**Marital status**		
Married	271	75.3
Other (Widowed or divorced or Single)	89	24.7
**Smoking status**		
Smoker	77	21.4
Non-smoker	283	78.6
**Use of alcohol**		
Yes	00.0	00.0
**Education level**		
Primary	50	13.9
Secondary	126	35.0
University	169	46.9
Postgraduate	15.0	04.2
**Occupation**		
Have a job	204	56.7
Jobless	66	18.3
Retired	90	25.0
**Monthly income**		
< 1000 NIS	101	28.1
1000 to < 2000 NIS	200	55.6
2000 to 3000 NIS	43	11.9
More than 3000 NIS	16	04.4
**NCDs types**		
Diabetes mellitus	102	28.3
Hypertension	128	35.5
Diabetes mellitus & hypertension	103	28.6
Other (Cancer or Asthma or CVD)	27	07.7
Complain NCDs/Years		
≤ 5 years	207	57.5
6 to 10 years	138	38.3
> 10 years	15	4.2

**Patients with NCDs received advice on health behaviors from healthcare professionals**: Table 2 displays the Mean percentages across the key recommendations of WHO-PEN Protocol 2 in the last twelve months, it reported as 54.5% ±13.6 in terms of taking regular physical activity, 49.3% ±11.5 in terms of eating a heart-healthy diet, 86.1% ±8.1 in terms of treatment adherence and attend regular follow up, and 43.9% ±16.8 in terms of stop tobacco for smokers' patients.

**Table 2:** Patients with NCDs that receiving advice to practice healthy behaviors

Key recommendations	NCDs patients (n=360)		

	Always & often N (%)	Sometimes N (%)	Rarely & never N (%)
**A. Take regular physical activity**			
Progressively increase physical activity to moderate levels (such as brisk walking); at least 150 minutes per week	32(8.9)	95(26.4)	233(64.7)
Control body weight and avoid overweight by reducing high calorie food and taking adequate physical activity	117(32.5)	166(46.1)	77(21.4)
**Mean Percentages ±SD:54.5 ±13.6**			
**B. Eat healthy diet**			
Restrict salt to less than 5 grams (1 teaspoon) per day	19 (5.3)	152(42.2)	189(52.5)
Reduce salt when cooking, limit processed and fast foods	162(45.0)	98 (27.2)	100(27.8)
Eat 5 servings (400–500 grams) of fruits and vegetable per day	68.0(18.9)	82 (22.8)	210(58.3)
Limit fatty meat, dairy fat and cooking oil (less than two tablespoons per day)	33 (9.2)	132(36.7)	195(54.2)
Replace palm and coconut oil with olive, soya, corn, rapeseed or safflower oil	65 (18.1)	100(27.8)	195(54.2)
Replace other meat with chicken (without skin)	45 (12.5)	81 (22.5)	234(65.0)
**Mean Percentages ±SD:49.3 ±11.5**			
**C. Treatment adherence**			
Teach how to take medications at home	349(96.9)	11 (03.1)	00.0(00.0)
Explain the difference between medicines for long- term control and medicines for quick relief	208(57.8)	143(39.7)	09(02.5)
Tell the reason for prescribing the medicine/s	306(85.0)	53 (14.7)	01 (0.3)
Show the appropriate dose	344(95.6)	16 (04.4)	00.0(00.0)
How many times a day to take the medicine	350(97.2)	10 (02.8)	00.0(00.0)
The need to take the medicines regularly as advised even if there are no symptoms	328(91.1)	28 (07.8)	04 (01.1)
**Mean Percentages ±SD:86.1 ±8.1**			
**D. For smoker only, Stop Tobacco (n=77)**			
Strongly advise all smokers to stop smoking and support them in their efforts	12 (15.6)	27 (35.1)	38 (49.4)
Assist in preparing a quitting plan	14 (18.2)	09 (11.7)	54 (70.1)
**Mean Percentages ±SD:43.9 ±16.8**			

Data are expressed as Mean Percentages ±SD for continuous variables and as percentage for categorical variables. M±SD: Mean Percentages ± Standard Deviation.

**Patients with NCDs received advice on taking regular physical activity**: The Mean percentages in terms of taking regular physical activity was reported as 54.5% ±13.6. Only 8.9% of the study participants claimed that they always or often received advice from healthcare professionals in the last twelve months to progressively increase physical activity to moderate levels (such as brisk walking); at least 150 minutes per week. Table 3 displays that the highest Mean percentages were reported in Khan Younis governorate with 58.1% ±12.2, followed by Rafah governorate with 56.8% ±13.1, and the lowest Mean percentages was reported in North Gaza governorate with 49.2% ±14.0. A statistically significant difference was found between the five Gaza strip governorates, Pvalue =.002. Table 4 displays statistically significant differences between Khan-Younis, Rafah governorates with North Gaza governorate with P value (.001,029) respectively.

**Table 3:** Patients with NCDs that received advice on health behaviors from healthcare professionals

Key recommendations		Take regular physical activity (n=360)		Eat healthy diet(n=360)		Adherence to treatment(n=360)		Stop Tobacco (n=77)	

Variables		Mean Percentages ±SD	*P* value	Mean Percentages ±SD	*P* value	Mean Percentages ±SD	*P* value	Mean Percentages ±SD	*P* value
**Area**	**North Gaza**	49.2±14.0	.002	44.8±12.6	.002	81.5±7.6	.001	41.1±12.7	.59
	**Gaza**	54.6±14.3		49.8±11.6		88.0±9.1		45.6±16.3	
	**Deir Al** **Balah**	55.0±11.5		48.8±9.8		86.9±7.6		44.4±14.2	
	**Khan** **Younis**	58.1±12.2		52.1±10.2		86.3±6.3		41.0±16.9	
	**Rafah**	56.8±13.1		50.9±11.1		87.1±5.9		42.2±14.1	

Data are expressed as Mean Percentages ±SD for continuous variables and as percentage for categorical variables. Mean Percentages ±SD: Mean Percentages ± Standard Deviation. The differences between means were tested by using one-way ANOVA

**Table 4:** Patients with that received advice on health behaviors from healthcare professionals by governorates

Key recommendations		Physical activity	Diet	Treatment
**(I) Area**	**(J) Area**	*P value*	*P value*	*P value*
**North Gaza**	**Gaza**	.063	.028*	.001*
	**Deir Al Balah**	.167	.456	.002*
	**Khan Younis**	.001*	.001*	.002*
	**Rafah**	.029*	.050*	.002*

The differences between means were tested by using one-way ANOVA, Bonferroni Post hock was used.

**Patients with NCDs received advice in terms of eating a heart-healthy diet**: Table 2 displays that the Mean percentages in terms of eating a heart-healthy diet was reported as 49.3% ±11.5. Only 5.3% of the study participants claimed that they always or often received advice from healthcare professionals in the last twelve months, to restrict salt to less than 5 grams (1 teaspoon) per day, 9.2% to Limit fatty meat, dairy fat, and cooking oil (less than two tablespoons per day), and 12,5% to replace other meat with chicken (without skin). Table 3 displays that the highest Mean percentages were reported in Khan Younis governorate with 52.1% ±10.2, followed by Rafah governorate with 50.9% ±11.1, and the lowest Mean percentages were reported in North Gaza governorate with 44.8%±12.6, followed by Gaza governorate with 49.8% ±11.6. A statistically significant difference was found between the five Gaza strip governorates, P-value =.002. Table 4 displays statistically significant differences between Khan-Younis, Rafah, and Gaza governorates with North Gaza governorate with P value (.001,050, .028) respectively.

**Patients with NCDs received advice in terms of treatment adherence**: Table 2 displays that the Mean percentages in terms of treatment adherence was reported as 86.1% ±8.1. It can be seen that the Mean percentages in terms of treatment adherence is the highest level of adherence among the key recommendations from the healthcare professionals based on the patients' views. 57.8% of the study participants claimed that they always or often received explaining the difference between medicines for long-term control and medicines for quick relief, and 85.0% telling them the reason for prescribing the medicine/s. Table 3 displays that the highest Mean percentages were reported in the Gaza governorate with 88.0% ±9.1, followed by the Rafah governorate with 87.1% ±5.9, and the lowest Mean percentages was reported in North Gaza governorate with 81.5% ±7.6. A statistically significant difference was found between the five Gaza strip governorates, Pvalue =.001. Table 4 displays statistically significant differences between Gaza, Deir Al Balah, Khan Younis, and Rafah governorates with North Gaza governorate with P-value <.05.

**Patients with NCDs received advice in terms of stop tobacco:** Table 2 displays that the Mean percentages in terms of stop tobacco was reported as 43.9% ±16.8. It can be seen that the Mean percentages in terms of stop tobacco is the lowest level of adherence among the key recommendations from the healthcare professionals based on patients' views. 15.6% of the study participants claimed that they always or often received strong advice to stop smoking and support them in their efforts, and 18.2% assisting in preparing a quitting plan. Table 3 displays that the highest Mean percentages were reported in the Gaza governorate with 45.6% ±16.3, followed by Deir Al Balah 44.4%±14.2, and the lowest Mean percentages was reported in Khan-Younis governorate with 41.0% ±16.9. There are no statistically significant differences between the five Gaza Strip governorates in terms of stop tobacco.

## Discussion

Globally, the four major NCDs (cardiovascular diseases (CVDs), cancers, chronic respiratory diseases, and diabetes) account for the majority of deaths. These four NCDs share common behavioral risk factors including tobacco use, unhealthy diet, physical inactivity, and alcohol use. There is an extensive scientific agreement that these factors contribute significantly to NCDs morbidity and mortality (1, 4).

Many studies were conducted by researchers worldwide and focus on healthcare professionals' adherence to counseling on healthy behaviors for NCDs patients from healthcare professionals' perspectives, as well as, from the patients' perspectives (14, 19, 20). In the Palestinian context, there are many studies conducted to investigate the healthcare professionals' adherence to educate healthy behaviors to patients with NCDs from healthcare professionals' perspective, but little is known whether patients with non-communicable diseases receive advice on health behaviors from healthcare professionals (10, 21). The current study aimed to explore whether patients with non-communicable diseases receive advice on health behaviors from healthcare professionals in the PHCs in the Gaza Strip, Palestine. The results of our study provided preliminary evidence for whether patients with noncommunicable diseases receive advice on health behaviors from healthcare professionals. The study demonstrated that the Mean percentages across the key recommendations of WHO-PEN were reported as 54.5% ±13.6 in terms of taking regular physical activity. 49.3% ±11.5 in terms of eating a heart-healthy diet. 86.1% ±8.1 in terms of treatment adherence and attend regular follow up. 43.9% ±16.8 in terms of stop tobacco for smokers' patients. Firstly, the highest Mean percentage was reported in terms of treatment adherence with (86.1%), which is consistent with previous study conduct among healthcare professionals in the Gaza strip which showed that 92.6% of health providers claimed that they sometimes or always adhere to counseling on health behavior in terms of treatment (13). The good news from our study is that healthcare professionals realize the importance of adherence to education the NCDs patients to treatment and regular follow-up. Secondly, the Mean percentage in terms of taking regular physical activity was reported as 54.5% ±13.6. In contrast, a study conducted in the Gaza strip among healthcare professionals demonstrated that 87.5% of health providers claimed that they sometimes or always adhere to counseling on health behavior in terms of physical activity (13). A study conducted in Canada showed that 42% of patients reported that they often or always receiving advice from their physicians on exercise (22). In Australia, 24.4% of participants reported that they received advice about exercise or physical activity from Physicians, the advice is most likely to be given to patients with chronic diseases which ranged from about 35% for a patient with one type of chronic disease to nearly 48% for a patient with two or more types of chronic diseases (23). Thirdly, the Mean percentage in terms of eating a heart-healthy diet was reported as 49.3% ±11.5. A study conducted in the Gaza strip among healthcare professionals demonstrated that 80.5% claimed that they sometimes or always adhere to counseling on health behaviors in terms of eating a heart-healthy diet (13). In Canada, around 38% of patients reported that they often or always receiving advice from their physicians on a healthy diet (22). Fourthly, the Mean percentage in terms of stop tobacco for smokers' patients was reported as 43.9% ±16.8. A study conducted in the Gaza strip among healthcare professionals demonstrated that around 55% claimed that they sometimes or always adherence to counseling on health behavior in terms of stop tobacco (13). In the USA, a study conducted demonstrated that healthcare providers advised patients to quit smoking ranged between 54.7% to 73.9% (24). Generally, the study demonstrated that there were many significant variations between the Gaza strip governorates in advising patients in terms of taking regular physical activity, eat a healthy diet, and treatment adherence. In terms of taking regular physical activity, Khan-Younis and Rafah governorates were the highest Mean percentages. In contrast, the North Gaza governorate was the lowest. Furthermore, in terms of eating a healthy diet statistically significant differences were found between Khan-Younis, Rafah, and Gaza governorates with North Gaza governorate. Besides, in terms of treatment adherence, statistically significant differences were found between all Gaza Strip governorates with North Gaza governorate. The result of this study is consistent with a previous study in Gaza strip which demonstrated that the North Gaza governorate centers were the lowest governorate centers ready to provide counseling on healthy behaviors with a readiness score of 56.3% (10). A possible explanation of the significant differences between the five Gaza Strip governorates that healthcare professional skills in some governorates are poorly developed or underused, inequitable distribution of health centers and healthcare professionals within Gaza Strip, favoring Gaza Strip central areas, contributes to health inequity (25, 26).

Possible limitations are this study is a cross-sectional study and use a convenience sample technique, and it asked about the advice without considering who provide this advice (Physician or Nurse). Besides, the study focused on advice only and did not observe the exact time spend in the advice sessions, and it is based on self-reported data, which could lead to recall bias.

In conclusion, Participants claimed that the vast majority of them had received advice from healthcare professionals regarding attending regular follow-up and treatment adherence. In contrast, participants reported receiving advice from health care professionals regarding regular physical activity, eating a healthy diet, and stopping tobacco are suboptimal. There is a need to develop a strategy to ensure that healthcare professionals are committed to providing advice on health behaviors for patients with non-communicable diseases. The results could help the decision-maker to plan for review the present way used by healthcare professionals to advising NCDs patients on healthy behaviors at the PHCs. Further future studies are required to confirm these findings.
